# Transcranial high-frequency random noise stimulation does not modulate Nogo N2 and Go/Nogo reaction times in somatosensory and auditory modalities

**DOI:** 10.1038/s41598-023-30261-3

**Published:** 2023-02-21

**Authors:** Koya Yamashiro, Koyuki Ikarashi, Taiki Makibuchi, Sayaka Anazawa, Yasuhiro Baba, Tomomi Fujimoto, Genta Ochi, Daisuke Sato

**Affiliations:** 1 Institute for Human Movement and Medical Sciences, Niigata University of Health and Welfare, Niigata, 950-3198 Japan; 2 Department of Health and Sports, Niigata University of Health and Welfare, Niigata, 950-3198 Japan; 3 Major of Health and Welfare, Graduate School of Niigata University of Health and Welfare, Niigata, 950-3198 Japan; 4 Research Fellowship for Young Scientists, Japan Society for the Promotion of Science, Graduate School of Niigata University of Health and Welfare, Niigata, 950-3198 Japan; 5 Field of Health and Sports, Graduate School of Niigata University of Health and Welfare, Niigata, 950-3198 Japan

**Keywords:** Neurophysiology, Physiology

## Abstract

Transcranial random noise stimulation (tRNS) of the primary sensory or motor cortex can improve sensorimotor functions by enhancing circuit excitability and processing fidelity. However, tRNS is reported to have little effect on higher brain functions, such as response inhibition when applied to associated supramodal regions. These discrepancies suggest differential effects of tRNS on the excitability of the primary and supramodal cortex, although this has not been directly demonstrated. This study examined the effects of tRNS on supramodal brain regions on somatosensory and auditory Go/Nogo task performance, a measure of inhibitory executive function, while simultaneously recording event-related potentials (ERPs). Sixteen participants received sham or tRNS stimulation of the dorsolateral prefrontal cortex in a single-blind crossover design study. Neither sham nor tRNS altered somatosensory and auditory Nogo N2 amplitudes, Go/Nogo reaction times, or commission error rates. The results suggest that current tRNS protocols are less effective at modulating neural activity in higher-order cortical regions than in the primary sensory and motor cortex. Further studies are required to identify tRNS protocols that effectively modulate the supramodal cortex for cognitive enhancement.

## Introduction

Transcranial random noise stimulation (tRNS) can modulate cortical activity and enhance motor and sensory functions^1^. Furthermore, these effects may persist following stimulation, suggesting therapeutic value for neurological and neuropsychiatric diseases. For instance, Terney et al.^2^ reported that brief tRNS of the primary motor cortex (M1) increased the local motor evoked potential amplitude, a manifestation of enhanced excitability, for at least 60 min post-stimulation. Subsequent studies have also reported that tRNS is more effective than transcranial direct current stimulation (tDCS) for increasing MI excitability^3,4^. In addition, tRNS applied to a specific primary sensory cortex can modulate local neural activity and improve the perception of the modality subserved by that region, including the perception of somatosensory^5^, auditory^6,7^, and visual^8–10^ inputs, both during and after stimulation. Collectively, these and related findings suggest that tRNS is a more effective method for cortical modulation than other forms of transcranial electrical stimulation (tES).

In contrast to sensorimotor function, these tRNS protocols appear to have little to no effect on higher-order cognitive functions such as two-digit addition^11^, working memory^12^, and response inhibition^13,14^. However, such studies have focused only on behavioral responses rather than neurophysiological measures such as event-related potentials (ERPs), so the mechanisms for these differential effects remain unclear. Therefore, the present study investigated the effect of tRNS on Go/Nogo response time (RT) and error rate as behavioral measures of response inhibition and on the associated ERPs as a measure of neural excitability in the stimulated brain region.

Inhibitory control, the ability to suppress a thought or motor response, is a crucial component of executive function in daily life^15,16^. The inferior frontal gyrus (IFG) and dorsolateral prefrontal cortex (DLPFC) are the significant contributors to inhibitory control of responses to somatosensory^17^, auditory, and visual stimuli^18^ in functional magnetic resonance imaging (fMRI) studies. However, Brevet-Aeby^19^ reported that tRNS applied to the DLPFC did not improve inhibitory response accuracy despite improved Go/Nogo RT, while Brauer et al.^14^ reported that neither 6-Hz tACS nor tRNS of the IFG influenced Go/Nogo performance (error rate and RT) during stimulation (online) and following stimulation (offline). These discrepancies in behavioral responses to transcranial stimulation of primary and supramodal cortices may be explained by differential effects on neural excitability; however, no study has investigated the effect of tRNS on the excitability of supramodal inhibitory brain region using ERPs. In our previous study, we measured the subtracted Nogo N2 ERP (mean Nogo trial waveform minus mean Go trial waveform) in the both somatosensory and auditory cortex^20^ and found a positive correlation between the Nogo N2 amplitude and Go/Nogo RT for the somatosensory modality^21^, suggesting that a tRNS-induced increase in supramodal Nogo N2 amplitude could improve both somatosensory and auditory Go/Nogo RTs. Therefore, we evaluated the effects of tRNS on both behavioral and neurophysiological function by recording ERPs during somatosensory and auditory Go/Nogo paradigms.

## Methods

### Participants

Sixteen healthy undergraduate university male students (20.6 ± 0.9 years, height 173.5 ± 3.9 cm) participated in this study. This sample size was larger than the minimum of 14 needed for 80% power and a significance level of 0.05 based on an effect size of 0.30. Written informed consent was obtained from each participant after a full explanation of the study objectives and methods. The study was conducted in accordance with the Declaration of Helsinki and approved by the ethics committee of Niigata University of Health and Welfare, Niigata, Japan (approval number: 18090).

### Random noise stimulation

High-frequency oscillatory current tRNS (100–640 Hz, 1.5 mA, 10-s rise and fall periods) was applied for 15 min using sponge-covered 5 × 5 cm rubber electrodes connected to a neuroConn DC-Stimulator (neuroConn GmbH, Ilmenau, Germany). We set the stimulation mode to “noise HF”, which is a random level of current generated for every sample (sampling rate 1280 samples/s). The random numbers are normally distributed, and the probability density function follows a bell-shaped curve^2,3^. Stimulating electrodes were placed bilaterally at F3 and F4 according to the international 10–20 EEG standard to stimulate the DLPFC^19^.

### Somatosensory and auditory stimulation

Somatosensory ERPs were elicited by constant current square-wave pulses (duration 0.2 ms) delivered to the second and fifth digits of the dominant hand by ring electrodes. At each digit, the anode was placed at the distal interphalangeal joint and the cathode at the proximal interphalangeal joint. The fifth digit was stimulated in the Go condition, and the second digit in the Nogo condition. Stimulus intensity at the fifth digit was fixed at three times the participant’s sensory threshold, and that at the second digit was adjusted so that the participant reported the same sensation intensity as at the fifth digit. These stimuli elicited no pain or other unpleasant sensations. Auditory ERPs were elicited by a pure tone delivered binaurally through headphones (60 dB sound pressure level, 50 ms duration, 10 ms rise time, and 10 ms fall time). A 1000 Hz pure tone was delivered for the Go condition and a 1500 Hz pure tone for the Nogo condition. These same stimulation conditions were used in our recent study^20^.

### Experimental paradigms

**Figure 1:**
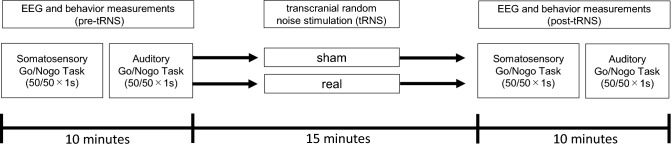
Experimental protocol. Sham and real tRNS sessions were pseudo-randomized in a single-blind design.

One week before electroencephalographic (EEG) measurements of ERPs, participants performed five practice sessions of both somatosensory and auditory Go/Nogo tasks, with each session consisting of 40 trials. A 1-min break was inserted between sessions to exclude the effects of short-term training. Figure 1 illustrates the experimental paradigm for EEG sessions. The actual study consisted of two separate sham and real tRNS sessions separated by at least one week to eliminate carry-over effects of stimulation, as reported in a previous study^13^. Sham and tRNS sessions order were randomized across participants. Participants performed separate somatosensory and then auditory Go/Nogo tasks on the same day, each consisting of 50 Go trials and 50 Nogo trials (i.e., equal 50% probabilities) presented in random order across sessions as described in our previous study^20^.

Individual somatosensory stimuli were presented at 2-s inter-stimulus intervals (ISIs) and auditory stimuli at 1-s ISIs to match the level of difficulty. In both somatosensory and auditory Go trials, participants were instructed to press a button as fast as possible using the dominant second digit when they perceived the Go stimulus (current or sound).

### EEG recording and analysis

ERPs were recorded using a SynAmps amplifier system (Neuroscan, El Paso, TX, USA) connected to five scalp electrodes (impedance < 5 kΩ) placed at Fz, Cz, Pz, F3, and F4 according to the 10–20 system and a reference placed on the left earlobe. The sampling rate was 1000 Hz, and EEG signals were recorded with a notch filter (50 Hz). According to our previous studies, trials with responses exceeding ± 100 μV were excluded from signal averaging. Signals were then band-pass filtered offline at 0.5–60 Hz. In both the somatosensory and auditory Go/Nogo paradigms, 50 artifact-free Go and 50 artifact-free Nogo trial waveforms were averaged pre- and post-stimulation (sham or tRNS) for each participant. Responses were analyzed from 100 ms before (baseline) to 500 ms after stimulus onset using Neuroscan 4.3 software. To extract Nogo potentials, we subtracted the averaged waveform of Go trials from Nogo trials as described in previous studies^22–25^. The subtracted waveform, termed the somatosensory or auditory Nogo N2 component, exhibited a negative peak relative to the prestimulus baseline at ~ 200 ms after the onset of the somatosensory or auditory stimulus. The peak latencies and amplitudes of Nogo N2 waveforms were measured between 120 and 250 ms after stimulus onset at 10–20 electrode positions Fz, Cz, Pz, F3, and F4 because this waveform is shown to reach a maximum around the frontal region for both somatosensory^21^ and auditory^20^ modalities.

### Data and statistical analysis

Behavioral data were obtained for Go/Nogo RT and commission error for the pre- and post-conditions in both the modalities. Parametric data (distribution confirmed using the Shapiro–Wilk test) were compared using paired-sample t-tests in both modalities. Nonparametric data were tested using Wilcoxon’s signed-rank test.

The neurophysiological data, Nogo N2 amplitudes for pre- and post-conditions in both modalities, were obtained. Parametric data were compared by two-way ANOVA with stimulation (pre vs post) and five electrode positions (Fz, Cz, Pz, F3, F4) as a within-participant factor. Nonparametric data were analyzed using Wilcoxon’s signed-rank test to compare the stimulation effects (pre vs post) by analyzing the pooled N2 amplitude of the five electrode positions.

For the two-way ANOVA analyses, we performed Mauchly’s sphericity assumption test; if it was violated, the Greenhouse–Geisser epsilon was used to correct the degrees of freedom. Statistical significance was set at p < 0.05 for all tests.

## Results

### DLPFC-targeted tRNS did not alter Go/Nogo RTs and commission error rates

All Go/NogoRT data were parametric while all commission error data were nonparametric. Therefore, paired-sample t-test and Wilcoxon’s signed-rank test were used to analyze Go/Nogo RT data and commission error rate data, respectively. The tRNS of the DLPFC did not significantly alter somatosensory Go/Nogo RT (235 ± 28 ms at baseline vs. 232.0 ± 30 ms post-stimulus, t (15) = 1.574, p = 0.136) or auditory Go/Nogo RT (254 ± 24 ms vs. 252 ± 27 ms, t (15) = 0.345, p = 0.734). Similarly, sham stimulation did not alter somatosensory Go/Nogo RT (232 ± 21 ms vs. 227 ± 21 ms, t (15) = 1.309, p = 0.210) or auditory Go/Nogo RT (252 ± 28 ms vs. 245 ± 21 ms, t (15) = 1.688, p = 0.112). Moreover, tRNS did not improve the somatosensory commission error rate (1.07% ± 1.60% at baseline vs. 1.03% ± 1.43% post-stimulation, p = 0.945) or auditory commission error rate (1.68% ± 1.71% vs. 0.92% ± 1.59%, p = 0.102). Sham stimulation also did not improve the somatosensory commission error rate (0.753% ± 1.04% vs. 0.459% ± 0.83%, p = 0.367) or auditory commission error rate (1.66% ± 1.75% vs. 0.98% ± 1.53%, p = 0.236).

### DLPFC-targeted tRNS did not alter somatosensory Nogo N2 amplitude

**Figure 2:**
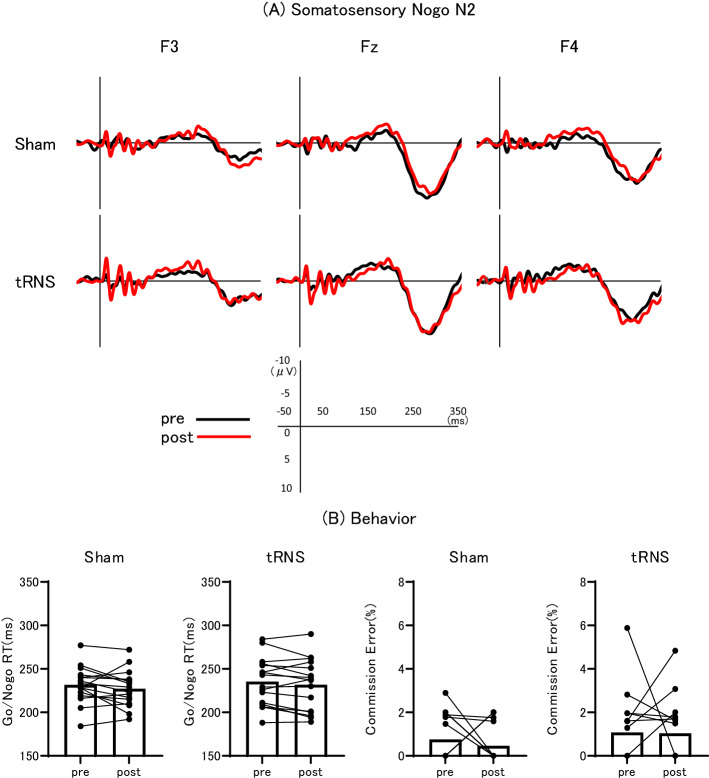
Grand-averaged somatosensory Nogo N2 waveforms and task performance. (**A**) Grand-averaged somatosensory Nogo N2 waveforms at electrode positions F3, Fz, and F4 before sham or tRNS (pre, black lines) and after sham or tRNS (post, red lines). (**B**) Within-subject and mean Go/Nogo reaction times (RTs, left panels) and commission error rates (right panels) before (pre) and after (post) sham and tRNS stimulation.

The peak amplitudes of somatosensory and auditory Nogo N2 waveforms at the five frontal and midline electrode sites Fz, Cz, Pz, F3, and F4 are summarized in Table 1, while Fig. 2A shows the grand-averaged waveforms of somatosensory Nogo N2 at three frontal electrode positions for the sham and tRNS sessions, and Fig. 2B summarizes somatosensory behaviors pre- and post-stimulation for sham and tRNS conditions. Somatosensory Nogo N2 amplitude was parametric data in both conditions. In the sham session, two-way ANOVA revealed no significant main effect of stimulation (F_(1, 15)_ = 2.065, p = 0.171), electrode (F_(2.093, 31.401)_ = 1.430, p = 0.255 ε = 0.523) and the interaction of stimulation × electrode (F_(2.545, 38.173)_ = 0.358, p = 0.751 ε = 0.636) on somatosensory Nogo N2 amplitude. In the tRNS session, two-way ANOVA also revealed no significant main effect of tRNS stimulation (pre- vs. post, F_(1, 15)_ = 0.122, p = 0.732 ε = 0.590) or electrode position (F_(2.358, 35.371)_ = 2.796, p = 0.066 ε = 0.590), and no significant real stimulation × electrode position interaction effect (F_(2.293, 34.401)_ = 0.328, p = 0.751 ε = 0.573) on somatosensory Nogo N2 amplitude.

**Table 1:** The peak amplitude ± SD of somatosensory and auditory Nogo N2.

Electrode	Somatosensory Nogo N2				Auditory Nogo N2			
	Sham		tRNS		Sham		tRNS	
	PRE	POST	PRE	POST	PRE	POST	PRE	POST
Fz	− 4.0 ± 3.1	− 4.7 ± 2.7	− 4.8 ± 2.9	− 4.9 ± 3.1	− 4.6 ± 2.4	− 3.7 ± 2.4	− 2.6 ± 3.8	− 3.1 ± 3.2
Cz	− 3.9 ± 3.6	− 4.9 ± 2.8	− 5.0 ± 2.1	− 5.2 ± 2.9	− 4.6 ± 2.8	− 4.1 ± 1.8	− 3.2 ± 2.8	− 3.4 ± 2.3
Pz	− 3.9 ± 3.1	− 5.3 ± 3.1	− 4.7 ± 2.1	− 5.4 ± 2.1	− 4.8 ± 1.8	− 4.2 ± 2.2	− 3.4 ± 2.1	− 3.7 ± 2.3
F3	− 2.7 ± 3.8	− 4.1 ± 2.5	− 3.6 ± 3.0	− 3.7 ± 2.6	− 4.5 ± 2.5	− 4.6 ± 2.0	− 1.8 ± 3.4	− 2.3 ± 3.1
F4	− 3.9 ± 2.7	− 4.9 ± 2.4	− 4.5 ± 3.0	− 4.5 ± 2.7	− 5.4 ± 2.5	− 3.9 ± 3.0	− 2.7 ± 3.3	− 3.0 ± 3.3

### DLPFC-targeted tRNS did not alter auditory Nogo N2 amplitude

**Figure 3:**
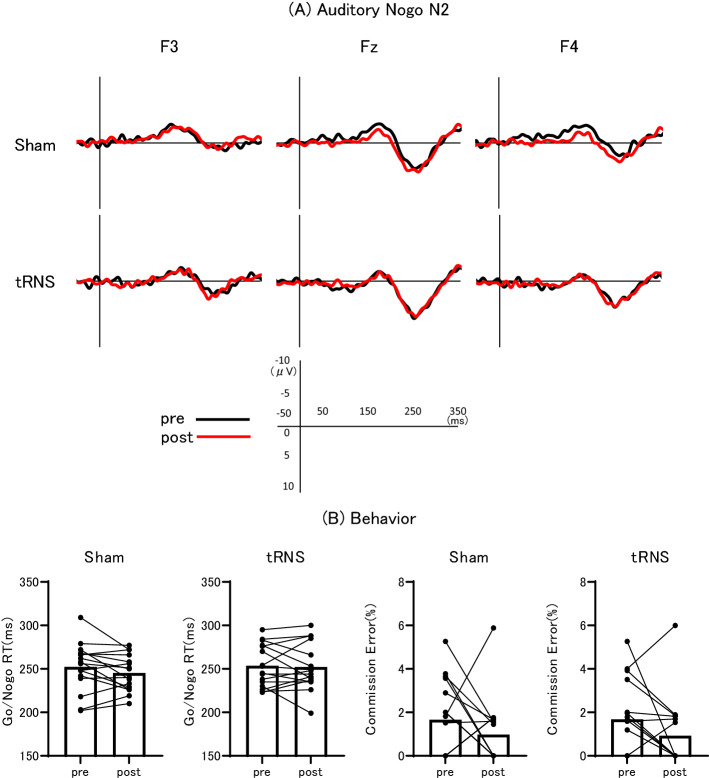
Grand-averaged auditory Nogo N2 waveforms and task performance. (**A**) Grand-averaged auditory Nogo N2 waveforms at electrode positions F3, Fz, and F4 before sham or tRNS (pre, black lines) and after sham or tRNS (post, red lines). (**B**) Within-subject and mean Go/Nogo reaction times (RTs, left panels) and commission error rates (right panels) before (pre) and after (post) sham and tRNS.

Figure 3A shows the grand-averaged waveforms of auditory Nogo N2 waveforms at three frontal electrode positions for sham and tRNS sessions, and Fig. 3B summarizes auditory behaviors pre- and post-stimulation for both sham and tRNS conditions. Auditory Nogo N2 amplitude was nonparametric data in both the conditions. Wilcoxon’s signed-rank test revealed no significant main effect of sham stimulation on auditory Nogo N2 amplitude (− 4.8 μV vs − 4.0 μV, p = 0.095). Similarly, Wilcoxon’s signed-rank test revealed no significant main effect of tRNS on auditory Nogo N2 amplitude (− 2.65 μV vs − 2.90 μV, p = 0.502).

## Discussion

The results of psychophysical stochastic resonance experiment revealed that noise stimulation for sensory area improved sensory detection performance in vision, audition, and touch^26^^.^ Longtin et al.^27^ reported the role of noise in neural encoding and the phase-locked response of sensory neurons to weak periodic stimulation. However, the effects of noise stimulation on the activity of higher-order cortical regions and behaviors are debated. Taking these reports into consideration, we investigated the effect of tRNS applied to the DLPFC on somatosensory and auditory Nogo N2 amplitude and Go/Nogo task performance but found no significant changes compared to sham stimulation. Therefore, we suggest that tRNS does not influence response inhibition because it does not appropriately modulate the DLPFC circuits contributing to this function.

tRNS is a relatively new brain modulation technique, and the underlying mechanisms are still relatively unexplored^1,28,29^. Transcranial stimulation is believed to modulate neural activity and behavior directly by facilitating or inhibiting neuronal firing, referred to as an online effect, and by inducing lasting changes in synaptic and circuit function (neuroplasticity) as an offline effect^29^. One potential online effect of tRNS is the repetitive opening of voltage-gated sodium channels and ensuing neuronal excitation^2,30^. Random noise may serve to boost weak signals, thereby enhancing processing fidelity, a phenomenon referred to as stochastic resonance^26,31^. We initially speculated that the offline effect of tRNS could modulate somatosensory and auditory Nogo N2 amplitudes and Go/Nogo RTs through such neuroplastic effects on DLPFC neurons but observed no such changes.

We propose several potential explanations for this lack of effect. The most parsimonious explanation is that tRNS does not alter the neuronal or circuit functions of supramodal cortices as effectively as it modulates sensory and motor cortex activities. Indeed, tRNS over the SI increased both S1 activity and gradient orientation discrimination task performance^5^. Similarly, tRNS over the auditory cortex increased the near-threshold stimulus detection rate in the temporal domain and reduced the peak latencies of P50 and N1 components, indicating accelerated auditory sensory processing^6^. Also, tRNS targeted at lateral occipitotemporal cortices enhanced face perception compared to motor cortex stimulation^9^.

Further, several studies have reported that tRNS effectively increase corticospinal tract excitability compared to tDCS^3,4^. Thus, tRNS may enhance excitability and oscillations more reliably when directed at sensory and motor regions through the induction of stochastic resonance^26^. Several recent studies have suggested that tRNS can modulate oscillations in the sensory cortex^6,7,32^, although not visual evoked potentials (VEPs) N1, N2, and P2 in the visual cortex^32^. Thus, tRNS to the supramodal cortex may have no electrophysiologically or behaviorally detectable effects.

Previous studies have suggested modest or no effects of tRNS targeted at the frontal cortex. For example, Bieck et al.^11^ found little to no effect of tRNS targeted at parietal and frontal cortices on two-digit addition, while Mulquiney et al.^12^ reported that tRNS of the left DLPFC did not improve working memory, although tDCS could. To our knowledge, only two behavioral studies have investigated the effect of tRNS on inhibitory control using the Go/Nogo paradigm, and the results were contradictory. Brauer et al.^14^ found that tRNS at 1 mA for 20 min over the unilateral IFG had no effect on Go/Nogo RT or commission errors, while Brevet-Aeby et al.^19^ found that tRNS applied to the bilateral DLPFC at 2 mA improved Go/Nogo RT but not commission error rate. However, neither study examined changes in ERPs or other neurophysiological indices of neural activity.

In contrast, Sallard et al.^13^ reported that tRNS applied over the bilateral IFG did not impact Go/Nogo performance, while concomitant MEG analysis revealed a significant increase in the beta band (20 Hz) spectral power following real tRNS. This finding suggests that tRNS may modulate cortical oscillations but not ERPs or VEPs, which reflect the summation of excitatory postsynaptic potentials and inhibitory postsynaptic potentials^33^. Therefore, tRNS may not affect synaptic activity in higher brain regions such as the DLPFC. However, future studies are required to assess if tRNS intensity, duration, timing, and (or) set number can modulate the activity of supramodal cortical regions in ways that improve higher brain functions.

In conclusion, tRNS appears to enhance oscillatory activity online in the primary sensory and motor cortex, thereby improving perception and motor task performance. However, the current tRNS protocol does not modulate synaptic activity offline in supramodal regions and thus fails to enhance higher brain functions.

## Data Availability

The datasets used and/or analyzed during the current study are available from the corresponding author upon reasonable request.
